# Does Biological Subtyping of Brain Structure in Schizophrenia Bring Us Personalized Psychiatry?

**DOI:** 10.1016/j.bpsgos.2026.100771

**Published:** 2026-07-08

**Authors:** Neeltje van Haren

**Affiliations:** Department of Child and Adolescent Psychiatry/Psychology, Erasmus University Medical Center, Rotterdam, the Netherlands

Biological subtyping in medicine means that instead of taking the symptoms as leading for disease definition, diseases are classified into distinct groups based on molecular, genetic, imaging or cellular characteristics. This would allow physicians to tailor targeted treatment to the specific needs and characteristics of an individual patient instead of to a diagnostic category or condition, an approach that is commonly referred to as personalized medicine (1).

Personalized psychiatry has been proposed as a promising approach for schizophrenia (2), which could support mental health professionals to move away from traditional trial-and-error treatment strategies. It may not only support subgroup classification with each subgroup having a preferred treatment but may also elucidate distinct pathophysiological or etiological pathways underlying different subgroups. Schizophrenia is heterogeneous, both clinically and biologically, with many of its neuroimaging findings being broadly distributed and highly overlapping across patients. This brain structural heterogeneity may reflect different discrete biological types of schizophrenia leading to similar alterations of mood or behavior within each subtype.

A personalized medicine approach brings several key clinical advances. First, different biological subtypes respond to different treatments. For example, neuromodulation may be the first choice for patients with specific structural brain deficits but not for others. Also, it may support mapping disease progression. If one neuroanatomical subtype is characterized by rapid cortical and subcortical volume reductions, it may signal a more aggressive form of the disease that requires early, intensive intervention. Furthermore, subgroups based on distinct neuroanatomical profiles may inform the development of entirely new classes of medication for mechanisms (e.g., excessive synaptic pruning or abnormal reward processing) that currently approved antipsychotics do not treat.

The work of Yu *et al.* (3), recently published in *Biological Psychiatry: Global Open Science*, originates from the concept of personalized psychiatry. In the schizophrenia research field, neuroimaging has been the most extensively used modality in efforts to identify biological subtypes of the disorder, Yu *et al.* (3) argue. In their review, they ask whether the accumulating literature on machine-learning subtypes of schizophrenia has actually demonstrated the existence of biologically meaningful categories, and they interrogate whether these clustering-derived entities are reproducible, clinically informative, or mechanistically coherent (3).

Their conclusions consist of 2 simultaneous truths. First, the subtyping approach has matured methodologically and now benefits from larger datasets, harmonized pipelines, and increasingly sophisticated computational approaches, although no standards exist. Second, despite this progress, the evidence for discrete, clinically relevant, and mechanistically informative neurobiological subtypes of schizophrenia remains surprisingly weak.

One of the strengths of the review is conceptual clarity. The authors distinguish dimensional decomposition methods from clustering approaches that assign individual patients to putative subgroups. This distinction is critical but frequently blurred in psychiatric neuroimaging. A proportion of the literature loosely describes biotypes, profiles, or latent patterns without specifying whether the underlying structure is continuous or categorical. By restricting their analysis to studies that produce individual patient-level subtype assignments using structural brain imaging data alone, the authors create a deliberately stringent test of the field’s central claim: Can structural brain measures identify biologically distinct forms of schizophrenia? The answer, according to this review, is not yet (3).

Across 18 studies, the same broad pattern of 3 clusters were reported, i.e., widespread abnormalities, regionally constrained abnormalities, and relatively preserved profiles. However, the specific anatomical regions implicated in these clusters vary substantially from study to study. Moreover, clinical correlates are inconsistent or absent. Where differences emerge, they generally reflect clinical severity gradients rather than qualitatively distinct symptom constellations. This is an important observation because it challenges a widespread implicit assumption in computational psychiatry that clustering algorithms necessarily reveal latent disease entities rather than partitions imposed on continuous variation. It leaves open the possibility that clinical and biological heterogeneity reflect the dimensional variability of a single illness with one common etiology, which varies in severity.

If the underlying biology is dimensional, overlapping, or hierarchical rather than categorical, clustering methods will still produce clusters because that is what clustering algorithms do. The resulting subtypes, estimated in a particular cohort of patients, can appear compelling, particularly when accompanied by visually striking distinct neuroanatomical maps, but reproducibility becomes elusive because the clusters reflect local optimization within noisy, high-dimensional data rather than stable biological entities. Yu *et al.* (3) are especially convincing when they show that inconsistency persists between cohorts even within the same algorithmic family. This observation undermines the explanation that divergent findings simply reflect methodological heterogeneity. Of course, clinical heterogeneity may still underlie these clustering inconsistencies; nevertheless, the implication is sobering: Current subtype solutions may be less robust than the field has hoped.

However, Yu *et al.* (3) avoid excessive pessimism. The article is not an argument against computational psychiatry or neuroimaging-based stratification, and the authors certainly do not draw a final conclusion. Rather, the article is a call for a higher evidential standard. In fact, the review arguably represents a sign of maturation in the field. The authors insist that reproducibility, external validation, longitudinal coherence, and clinical relevance must all be demonstrated before subtype claims are accepted (3). Few studies reported in Yu *et al.* (3) included rigorous external validation or longitudinal assessment. The proposed benchmarking standards—harmonized preprocessing, confound control, stability analysis, independent replication, and longitudinal consistency—should become minimum expectations in the biological subtyping field [also see (4)].

Alternative approaches, such as clustering based on multiple modalities, are also provided by Yu *et al.* (3) as a potential way to move forward, possibly yielding a more comprehensive understanding of brain-related clusters than any single modality provides alone. However, integrative models require stronger theoretical grounding; larger multimodal feature sets do not automatically produce more meaningful biology. Moreover, Yu *et al.* (3) rightly note that multimodal approaches introduce substantial interpretive and methodological complexity. Particularly, combining modalities beyond neuroimaging features sets, including cognition, genomics, and clinical data, can easily produce clusters dominated by whichever domain has the highest variance or strongest measurement reliability. Nonetheless, it may ultimately be unrealistic to expect structural neuroimaging alone to resolve psychiatric heterogeneity. Schizophrenia, or any other complex disease, is unlikely to respect disciplinary boundaries between anatomy, cognition, immune function, and environment.

Another potentially promising approach is to cluster disorder subtypes based on longitudinal brain measures. Instead of evaluating brain scans at a single point in time, longitudinal brain change as input allows models to capture clusters in temporal trends, such as the rate and shape of structural changes [e.g., (5)]. Yu *et al.* (3) only reported on one study in their review (6), that included longitudinal measures of brain structure, but only to examine treatment response to antipsychotic medication and transcranial magnetic stimulation in subtypes based on cross-sectional brain structural features. To further increase the complexity, the joint analysis of multimodal longitudinal features in an attempt to explore co-occurring trajectories among markers indicative of, e.g., brain volume atrophy, reduction of white matter integrity, and cognitive decline in individuals may be the way to move forward but will be particularly challenging [e.g., (7)].

To conclude, neuroimaging-based subtyping is often justified by the promise of precision psychiatry. However, precision medicine requires categorizing subtypes that improve outcome prediction or treatment selection or, at minimum, provide mechanistic understanding of the disorder. Most studies reviewed in Yu *et al.* (3) did not demonstrate clinically meaningful separation between brain-based subtypes of schizophrenia. Even when differences between clusters existed, they frequently reflected quantitative severity rather than qualitative distinction. This matters because severity gradients do not necessarily justify new nosological entities. A biologically informed classifier that merely distinguishes between patients with less versus more severe clinical profiles may still have prognostic utility, but it is conceptually different from identifying distinct disease mechanisms within schizophrenia. Nevertheless, future work on clustering approaches grounded in stronger biological evidence, including multimodal and longitudinal feature sets, and more rigorous validation standards may still help identify clinically and mechanistically meaningful heterogeneity within schizophrenia. Ultimately, to achieve meaningful clinical impact, the field may need to shift its focus from asking whether structural brain measures can identify biologically distinct forms of schizophrenia to asking whether such forms can be identified using multimodal and longitudinal patient data. However, given the substantial challenges associated with this endeavor, it remains uncertain whether the answer to this question will ultimately be an unequivocal yes.
